# Substantial Proton Ion Conduction in Methylcellulose/Pectin/Ammonium Chloride Based Solid Nanocomposite Polymer Electrolytes: Effect of ZnO Nanofiller

**DOI:** 10.3390/membranes12070706

**Published:** 2022-07-13

**Authors:** John Ojur Dennis, Abdullahi Abbas Adam, M. K. M. Ali, Hassan Soleimani, Muhammad Fadhlullah Bin Abd. Shukur, K. H. Ibnaouf, O. Aldaghri, M. H. Eisa, M. A. Ibrahem, Abubakar Bashir Abdulkadir, Vipin Cyriac

**Affiliations:** 1 Department of Fundamental and Applied Sciences, Universiti Teknologi PETRONAS, Seri Iskandar 32610, Perak, Malaysia; jdennis100@gmail.com (J.O.D.); hassan.soleimani@utp.edu.my (H.S.); mfadhlullah.ashukur@utp.edu.my (M.F.B.A.S.); abubakarbashir150@gmail.com (A.B.A.); 2 Centre of Innovative Nanoscience and Nanotechnology (COINN), Universiti Teknologi PETRONAS, Seri Iskandar 32610, Perak, Malaysia; 3 Department of Physics, Al-Qalam University Katsina, Katsina 820252, Nigeria; 4 Department of Physics, College of Science, Imam Mohammad Ibn Saud Islamic University (IMSIU), Riyadh 13318, Saudi Arabia; khiahmed@imamu.edu.sa (K.H.I.); odaghri@imamu.edu.sa (O.A.); mhsalim@imamu.edu.sa (M.H.E.); 9@imamm.org (M.A.I.); 5 Department of Physics, Manipal Institute of Technology, Manipal Academy of Higher Education, Manipal 576104, Karnataka, India; vipin.cyriac@learner.manipal.edu

**Keywords:** NCSPEs, dielectric properties, stability window, relaxation time, transference number

## Abstract

In this research, nanocomposite solid polymer electrolytes (NCSPEs) comprising methylcellulose/pectin (MC/PC) blend as host polymer, ammonium chloride (NH_4_Cl) as an ion source, and zinc oxide nanoparticles (ZnO NPs) as nanofillers were synthesized via a solution cast methodology. Techniques such as Fourier transform infrared (FTIR), electrical impedance spectroscopy (EIS), and linear sweep voltammetry (LSV) were employed to characterize the electrolyte. FTIR confirmed that the polymers, NH_4_Cl salt, and ZnO nanofiller interact with one another appreciably. EIS demonstrated the feasibility of achieving a conductivity of 3.13 × 10^−4^ Scm^−1^ for the optimum electrolyte at room temperature. Using the dielectric formalism technique, the dielectric properties, energy modulus, and relaxation time of NH_4_Cl in MC/PC/NH_4_Cl and MC/PC/NH_4_Cl/ZnO systems were determined. The contribution of chain dynamics and ion mobility was acknowledged by the presence of a peak in the imaginary portion of the modulus study. The LSV measurement yielded 4.55 V for the comparatively highest conductivity NCSPE.

## 1. Introduction

Researchers have recently shown great interest in developing polymer electrolytes (PEs) with specific properties, such as high ionic conductivity at ambient temperature, biodegradability, excellent mechanical properties, and robust performance in lightweight and thin energy storage systems [1]. Gel polymer electrolytes (GPEs) are one focus of research, due to their broad potential window and high ionic conductivity (close to that of liquid electrolytes) [2]. However, the fragile mechanical properties, internal short circuits, and restricted operating temperature of GPEs limit their practical applications in batteries and supercapacitors [2,3]. Solid polymer electrolytes (SPEs) have recently become extremely interesting because of their feasibility in high energy density and high power density devices [4].

Cellulose is the most abundant biological macromolecule that serves as a green polymer. Cellulose is insoluble in water and many other solvents, which limits its application in polymer sciences. However, cellulose acetate (CA), methylcellulose (MC), carboxymethyl cellulose (CMC), etc., are common cellulose derivatives, mostly used to prepare cellulose-based polymer electrolytes (PEs). Particularly, MC is extensively studied as a polymeric material due to its low cost, green characteristics, transparency, and excellent film-forming ability [5,6,7]. Besides, plant-based pectin (PC) is another biopolymer made from natural products, such as apples and berries, which are biodegradable and ecologically beneficial. In contrast to other polymers with bigger sizes and larger molecules connected to them, biopolymer pectin functions as a home, in which ions can move conveniently by elongating, owing to its smaller size and structural properties [8,9].

Whereas SPEs based on a single host polymer suffer from low ionic conductivity, polymer blending [10], incorporation of additives [11], polymer functionalization [12], and polymer crosslinking [13] are effective strategies to improve the performance of SPE systems. Specifically, polymer blending, a strategy of mixing two polymers to improve the amorphous phase of the polymer electrolyte systems, has been an excellent approach to improving the ionic conductivity of PEs. This technique offers a good channel in the polymer host matrix for fast ion mobility [14,15,16]. Furthermore, the combination of polymers offers more complexation sites and improves the amorphous phase for increased ions mobility, thereby increasing ionic conductivity [17,18]. Nevertheless, while blended polymer composite electrolytes have recorded appreciable ionic conductivity, the ionic conductivity value of these electrolytes does not meet the requirements of electrochemical devices. Salt diffusion is, therefore, required to achieve significant ionic conductivity. Proton-conducting PEs have demonstrated their candidacy in electrochemical devices, such as sensors, batteries, and supercapacitors [19]. Among protonous salts, ammonium salts are especially possible as alternatives to widely employed lithium salts, which are both expensive and toxic. Furthermore, ammonium salts also have low lattice energies and are substantial proton donors in the polymer matrix [20]. 

Scientists have recently shown a strong interest in nanocomposite polymer (NCP) films, owing to their spectacularly distinct nature and high potential for physical and electrical characteristics [21]. By doping an appropriate amount of nanofillers in the host polymer, the polymer system’s electrochemical characteristics can be significantly affected. In this regard, NCP films have been used in various possible applications, including secondary batteries, electrochemical cells, supercapacitors, and biosensors [22]. Among the most versatile metal oxide nanoparticles, zinc oxide nanoparticles (ZnO NPs or ZNs) stand out for their several exceptional electrical and optical properties [23,24]. In addition to having piezoelectric capabilities, ZnO is an n-type semiconductor with a 3.37 eV wide bandgap [25]. As a result of these characteristics, ZnO may be used in a wide range of sophisticated applications, including the construction of microelectronic circuits, solar energy conversion, sensors, and electrochemical devices [25,26,27,28]. Recently, ZN has been disseminated into numerous SPE systems in order to increase ionic conductivity, microstructure, and mechanical characteristics of the resulting NCSPEs [29,30,31]. Moreover, ZN is a widely used filler that is economical and more readily available than other metal oxide nanoparticles [23,32].

Herein, we report on the synthesis of proton ion-conducting NCSPEs comprising MC-PC blend as a host material for ion transport and ammonium chloride (NH_4_Cl) as a proton donor. The weight ratios of NH_4_Cl and ZN were varied to investigate the effect of an ammonium salt and ZnO nanoparticles on ionic conductivity, dielectric behaviour, ion transference number, and potential window of the SPEs. The SPEs were prepared via an ultrasonication-assisted solution casting technique. Ultrasonication was employed to speed up the precursor solution’s preparation and ensure the homogeneity of the solution. To the best of our knowledge, there have been no reported articles on proton ion conducting SPEs based on a MC/PC blend. Therefore, this work will serve as new material for highly transparent SPEs.

## 2. Materials and Methods

MC (viscosity of 4000 cP) was purchased from Sigma Aldrich, Malaysia, PC (M_w_ of 30,000 gmol^−1^), K_3_PO_4_ (M_w_ of 212.27 gmol^−1^), glycerol (M_w_ of 92.09 gmol^−1^) and ZnO nanoparticles (~10 nm) were purchased from R and M Chemicals. All chemicals used were supplied by Evergreen Chemicals Supply (Selangor Malaysia) and were used without any treatment. DI water was used as the only solvent throughout the experiment. 

### 2.1. Preparation of Electrolyte Samples

**Figure 1:**
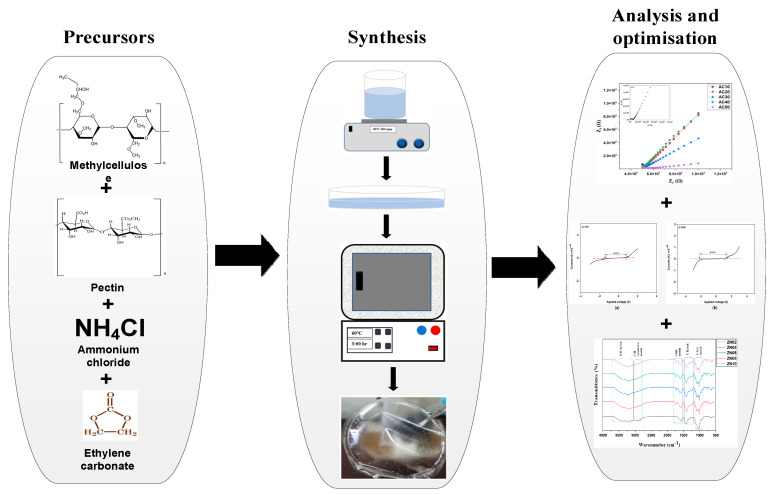
Schematic representation of NCSPE synthesis and optimisation.

Firstly, known concentrations of MC (30 wt.%) and PC (70 wt.%) were individually dissolved in DI water (20 mL and 60 mL, respectively) until fully dispersed. To obtain the MC/PC blend solution, the individual MC and PC solutions were mixed and stirred at room temperature for a few hours. The solution was ultrasonically shaken for 30 min to obtain a homogenous solution. Various NH_4_Cl (0–50 wt%) concentrations were added to the solution and stirred for some time to prepare the NH_4_Cl doped samples before they were cast on a petri dish. The cast solution was dried in an oven at 50 °C for two days to obtain the solid electrolyte films. The prepared films were peeled off the petri dish and stored in a desiccator containing silica gel. To prepare the NCSPEs, different amounts of ZnO NP (2–10 wt.%), initially dissolved in 2% acetic acid were incorporated into the solution containing MC, PC and NH_4_Cl and were stirred for a few minutes, followed by ultrasonication to ensure homogeneity. The prepared solution was cast in a petri dish and oven-dried, as explained earlier. To easily identify samples, AC00, AC10, AC20, AC30, AC40 and AC50 were assigned to samples containing 0 wt.%, 10 wt.%, 20 wt.%, 30 wt.%, 40 wt.% and wt.% NH_4_Cl, respectively. Similarly, NCPEs containing 2 wt.%, 4 wt.%, 6 wt.%, 8 wt.% and 10 wt.% ZnO NPs were labelled ZN02, ZN04, ZN06, ZN08 and ZN10. The schematic representation of NCSPEs synthesis, analysis and optimization is depicted in Figure 1.

### 2.2. Characterization of Electrolyte Samples

Fourier transform infrared (FTIR) analysis was used to examine the functional groups of the produced samples and their related band shifts. The FTIR analysis was performed in the 4000–400 cm^−1^ region with KBr pellets and a scanning resolution of 4 cm^−1^ using an FTIR spectrometer (Bruker Instruments, model Aquinox 55, MA, USA). Scanning electron microscopy (SEM, Zeiss EVO LS15, Bremen, Germany) was used to study the surface morphology of the prepared samples. Electrochemical studies were carried out to ascertain the PE’s ionic conductivity, dielectric relaxation, and operating potential window of the SPEs and NCSPEs. The measurement was performed using the AUTOLAB/AUT51018 in a two-electrode setup. Each sample was sandwiched between two blocks of a stainless-steel sample holder with a 3.142 cm^2^ surface area that were positioned opposite each other. 

## 3. Results and Discussion

This section is divided by subheadings. It should provide a concise and precise description of the experimental results, and their interpretation, as well as the experimental conclusions that can be drawn.

### 3.1. FTIR Analysis

**Figure 2:**
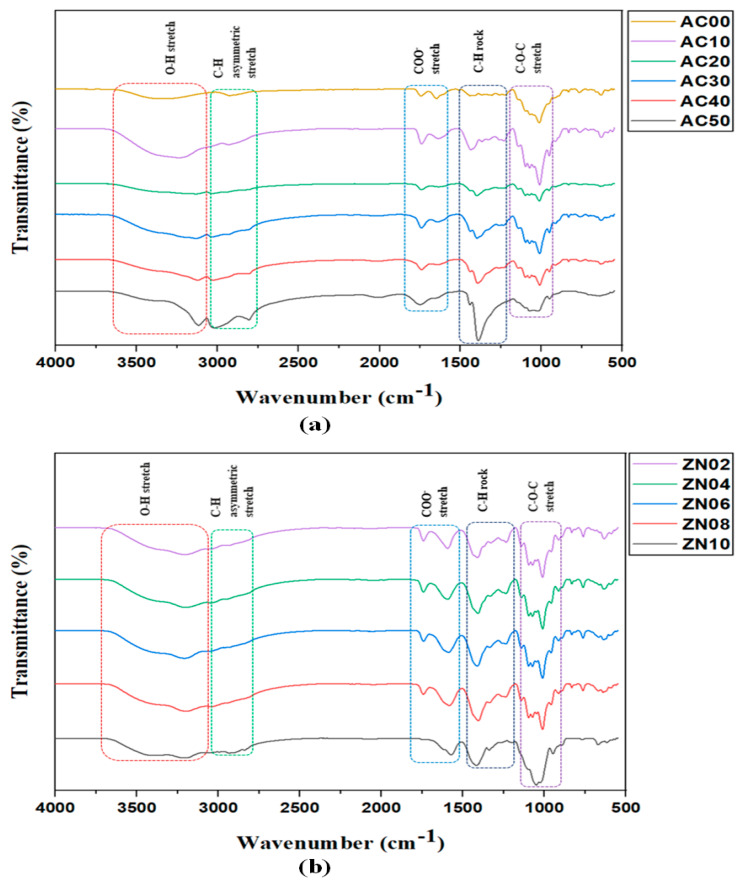
FTIR spectra at 500 to 4000 cm^−1^ for (**a**) MC/PC/NH_4_Cl SPE (**b**) MC/PC/NH_4_Cl/ZnO NCSPE.

FTIR has distinguished itself as a well-established and highly proficient technique for studying changes in the local structure of polymers at the molecular level. According to Jothi et al. [33], the intensity fluctuation and the identified band shifting in the FTIR spectrum in a salt-plasticizer complexed system shows that the salt and plasticizer have been completely complexed and dissolved in the polymer mix. Complexation may be linked to changes in the infrared spectrum, such as band shifting and the introduction or extinction of an infrared spectrum band. Thus, a comparison of the infrared spectra of various materials may be used to reveal the complicated composition of the polymer matrix [34]. Therefore, this work relied heavily on the alteration of FTIR spectral band assignments to explain polymer-salt complexation and their interactions with the nanofiller. The FTIR spectra of MC/PC/AN SPEs and MC/PC/AN/ZN NCSPEs (between 4000 and 500 cm^−1^) are shown in Figure 2a,b, respectively. From the FTIR spectra, the following observations can be drawn:

#### 3.1.1. Confirmation of MC/PC Backbone 

According to the literature, the FTIR spectrum of pristine MC exhibits characteristic bands in the vicinity of 3250 and 2810 cm^−1^ belonging to O-H stretching, and C-H asymmetric stretching, respectively. Other spectral bands reported are 1668, 1458 and 1058 cm^−1^ which correspond to COO^−^ asymmetric stretching, C-H rocking and C-O-C stretching MC, respectively [35,36,37]. The tiny peaks around 946 cm^−1^ are the Infrared spectra characterization for MC [16]. For pristine PC film, the FTIR prominent bands are found within the proximity of 3334, 2910, 1652, 1422 and 1099 cm^−1,^ which belong to O-H stretching, C-H stretching, COO^−^ stretching, C-H bending, and C-O-C stretching of PC, respectively. Additional bands found at 1740, 1228, and 1010 cm^−1^ are typical of -COOCH_3_ stretching_,_ O-H bending and -CH-O-CH- stretching of PC, respectively [37,38,39,40].

**Figure 3:**
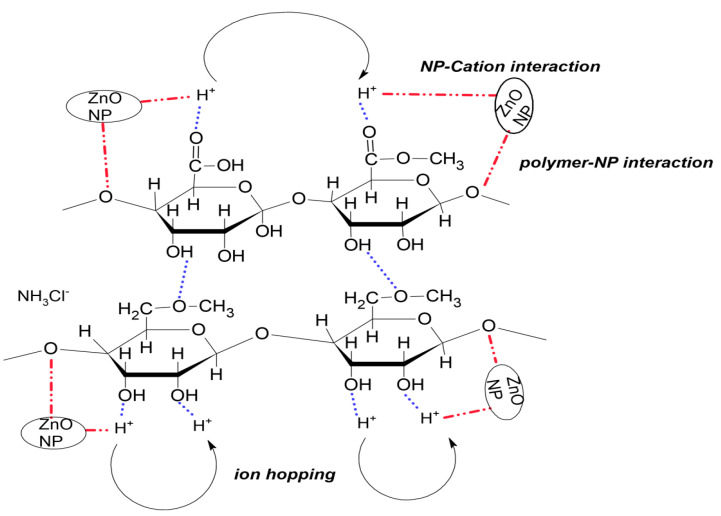
Probable interaction scheme of NH_4_Cl salt and ZnO nanofiller with MC/PC based polymer blend.

In this work, the FTIR of MC/PC blend was investigated by analysing band shifts in the blend system. Upon mixing MC with PC, the synthesised polyblend film exhibited a shift in IR spectrum relative to conventional peaks of pristine MC and PC films, due to the successful blending of MC with PC. The prominent O-H stretch was observed at about 3370 cm^−1^, due to hydrogen bond interaction between the covalently bonded H atom of one polymer and the adjacent polymer’s covalently bonded oxygen atom. From Figure 3, it can be seen that the hydrogen atom of the hydroxyl band (O-H) in MC interacted with the glycosidic linkage (C-O-C) band in pectin to form the MC/PC polymer blend. Other band assignments found in the MC/PC polyblend films created due to shifting in the IR band spectrum were C-H stretching (2931 cm^−1^), -COOCH_3_ stretching (1726 cm^−1^), COO^−^ stretching (1615 cm^−1^), C-H bending (1438 cm^−1^), O-H bending (1216 cm^−1^), C-O-C stretching (1066 cm^−1^), -CH-O-CH- stretching (1003 cm^−1^) and =C-H bending (949 cm^−^^1^).

#### 3.1.2. Confirmation of Complexation with NH_4_Cl

It can be inferred from Figure 2a that doping the MC/PC blend with 10 to 50 wt.% NH_4_Cl caused some alterations of the IR spectrum band. As seen, the presence of NH_4_Cl made the OH band of the MC/PC blend weaker. Initially, the transmittance intensity of the OH band increased when 10 wt.% NH_4_Cl was added. However, as the salt concentration increased, this band broadened further until a small sharp peak appeared at 3126 cm^−1^, possibly due to the aggregation of excess ammonium salt-forming salt agglomerates in the polymer matrix. This resulted in the production of ion pairs that did not contribute to ionic conduction and lowered the ion density [41]. This shift confirmed that complexation between the MC/PC mix and NH_4_Cl had occurred. The more NH_4_Cl was added to the MC/PC mix, the greater the concentration of H^+^, and, thus, more electrons were pulled towards MC through C-O to form hydrogen bonds. The interaction of the conducting species (H^+^) formed by NH_4_Cl increase the number of mobile ions, thereby increasing the electrolytes’ conductivity. These FTIR data, therefore, demonstrated that the oxygen atoms in the functional groups acted as complexation sites for the salt’s cation, resulting in the formation of a dative bond.

The C-H bond found at 2931 cm^−1^ in the MC/PC film weakened as the concentration of NH_4_Cl increased up to 40 wt.% (AC40). This confirmed that NH_4_Cl salt disrupts the MC/PC host polymer backbone. As the concentration of NH_4_Cl increased to 50 wt.% (AC50), C-H peaks became evident with the protrusion of a new height at around 2728 cm^−1^, probably due to the aggregation of H^+^. Since complexation of MC/PC with NH_4_Cl was considered to occur at the C=O peak, owing to the existence of a lone pair of electrons that attract free ions, raising the ammonium salt concentration was found to cause a decrease in the transmittance intensity (and also a shift in the bandwidth of the doped samples to the lower wavelength area) of the COO^-^ stretch. This apparent decrease in transmittance strength and shifting of bands indicated the development of complexes between salt cations and functional groups in the polymer blend matrix and demonstrated that the MC/PC host mix polymer and the added salt had significant interaction. A similar result was obtained by Nadirah et al., [16] showing a decrease in MC/PAN blend intensity. 

#### 3.1.3. Confirmation of ZnO NP Incorporation

A careful examination of the FTIR spectra of the prepared NCSPEs reveal that the spectrum of each ZnO NPs incorporated in MC/PC/NH_4_Cl film closely resembled that of MC/PC/NH_4_Cl film. However, the intensities of a few peaks varied significantly with increasing ZnO NPs concentrations, with only a slight shift in the positions of some of the peaks. The alterations in peak intensities strongly suggested that ZnO NPs interact with some functional groups of MC/PC/NH_4_Cl films. A broad OH band was observed between 3194 to 3677 cm^−1^ for all the samples containing ZnO NPs (Figure 2b). The addition of ZnO nanofillers revealed a decrease in the peak intensity. Moreover, the stretching of the OH band peak caused by ZnO nanofillers might be attributed to water molecules absorbed by ZnO NPs. The presence of OH on the surface of ZnO nanofillers facilitated Lewis’s acid-base base interaction, producing an alternative conduction channel for ions in the polymer matrix. Consequently, the polymer electrolyte incorporated with ZnO NPs had greater ionic conductivity. This is seen in the EIS results of ZnO nanofiller incorporated films (ZN02–ZN10). When an excessive amount of nanofiller was introduced to the electrolyte, fewer cations interacted with the OH group of pectin in the MC/PC blend due to their small size, which allowed them to recombine with an anion to produce neutral ion pairs.

The C-H asymmetric stretching of nanofiller incorporated NCSPEs were found at ~2912 cm^−1^. For all NCSPEs, the IR band due to C-H stretch diminished except for ZN10. This could be due to the plasticizing influence of ZnO nanofiller. The C–O–C symmetric stretching mode of both MC and PC in the MC/PC mix gave clear evidence of complexation through band shifting due to the presence of nanofillers. Due to the interaction of the nanofiller’s surface acid group with the Lewis base upon adding ZnO nanofiller, the polymer salt matrix also played a significant role in polymer–ion and polymer–ion–nanofiller interactions in the NCSPEs. Ammonium chloride-anion and the surface groups of ZnO nanoparticles also exhibited the Lewis acid-base interaction within the polymer matrix. This mechanism decreased the Coulombic interaction between the salt’s cation and anion, increasing the NH_3_Cl^−^ dissolvability, and, thus, more cations were available to coordinate with the oxygen atoms in the C-O-C groups of the MC/PC blend. Further nanofiller addition resulted in a shift in the transmittance intensity’s band location for the stretching mode.

A minor OH bending was seen at the vicinity of 3250 cm^−1^ in the AC50 sample owing to the interaction of NH4Cl with the MC/PC film. With the introduction of ZnO nanofillers, this band seemed to expand appreciably. In general, all the changes observed in the FTIR spectrum were apparent indications that the salt and nanofiller had been effectively complexed and dissolved in the polymer blend.

### 3.2. DSC Thermogram

**Figure 4:**
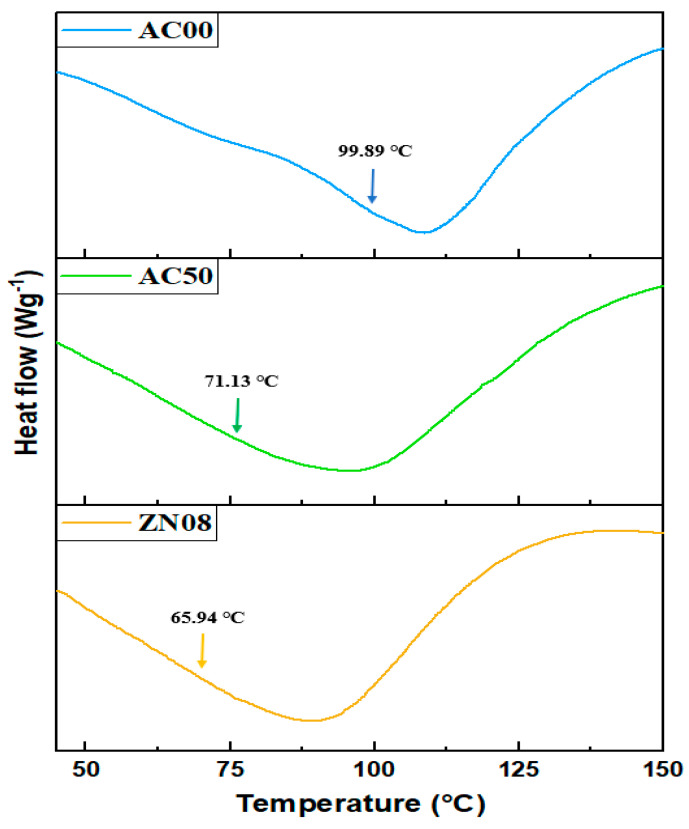
DSC thermograms for some selected samples.

In SPEs, the polymer–salt complexation promotes polymer backbone flexibility by reducing the intermolecular interaction within the composite polymer host. This feature enables rapid segmental motion, which aids in ion migration [42]. Additionally, the ionic mobility of polymer chains is dependent on their segmental motion in the amorphous phase, which is defined by the glass transition temperature (T_g_). The T_g_ value indicates the temperature at which a material’s glassy phase transforms into a rubbery phase [20]. Figure 4 shows the T_g_ of NCSPEs obtained from DSC curve. Each thermogram demonstrates a single-step transition of an endothermic process, proving the miscibility of MC with PC and their complexation with NH_4_Cl. It can be observed that T_g_ of the MC/PC blend decreased from 99.89 °C to 71.13 °C upon the incorporation of ammonium salt. The decrease in T_g_ readings as the temperature decreased suggested that the polymer salts segment became less firm in its amorphous phase structure. Additionally, the decreasing T_g_ values of the polymer host were associated with an increase in the ionic conductivity of the polymer electrolytes. 

The addition of salt to the MC/PC mix weakened the dipole–dipole interactions between the MC/PC chains, allowing the ions to easily travel across the polymer chain network in the presence of an electric field, hence increasing the conductivity. In addition, the introduction of adequate quantities of ZnO nanofillers reduced the T_g_ of the MC/PC-based electrolyte because the combination of salts and nanofillers promoted the amorphous region of the polymer electrolytes and boosted the ionic transport inside the polymer matrices [43].

### 3.3. SEM Analysis

**Figure 5:**
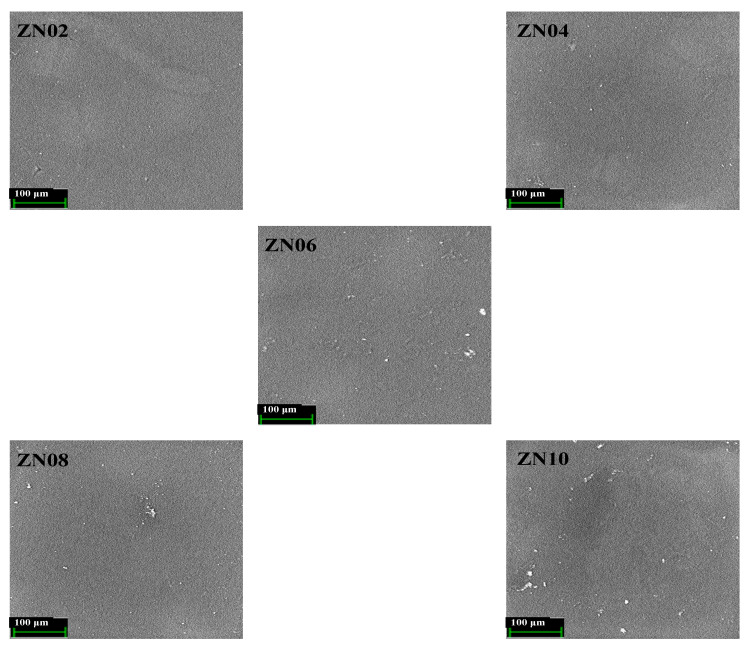
SEM images of NCPEs.

After incorporating ZnO NP into AC05, the electrolyte samples (ZN02, ZN04, ZN06, ZN08, and ZN10) were submitted to SEM examination to investigate the morphology and dispersion of the NPs in the MC/PC/NH4Cl polymer matrix. Typical SEM data samples are provided in Figure 5, where it is obvious that the ZnO NPs were evenly dispersed throughout the polymer. All of the prepared NCPEs had smooth surfaces with no apparent defects. This implied that MC and PC were compatible, as well as that ZnO NPs dispersed ammonium salt adequately. However, when the quantity of ZnO NP in the sample increased, agglomerates formed here and there on the surface of the NCPE. These agglomerates increased as the concentration of ZnO increased and might be attributable to salt recrystallization generated by an excess of ZnO NPs.

### 3.4. Ionic Conductivity Studies 

Electrochemical impedance spectroscopy (EIS) has been used extensively in the investigation of electrochemical behaviour and ion transport in a range of ionic materials, including electrodes and SPEs. Using this method, impedance spectra for the SPE films were generated and analyzed [44,45]. All impedance spectra observed in this study showed a prominent spike at low frequency, due to double layer capacitance which existed at the interface of the electrode and the electrolyte sample. A closer look at the impedance spectra showed a tiny semicircle at the high frequency region of most of the samples. This was attributed to the bulk resistance (R_b_) of the electrolyte samples [44]. Here, the value of R_b_ was obtained by measuring the point at which the semicircle touched the real impedance axis (Z_r_), just before the spike.

**Figure 6:**
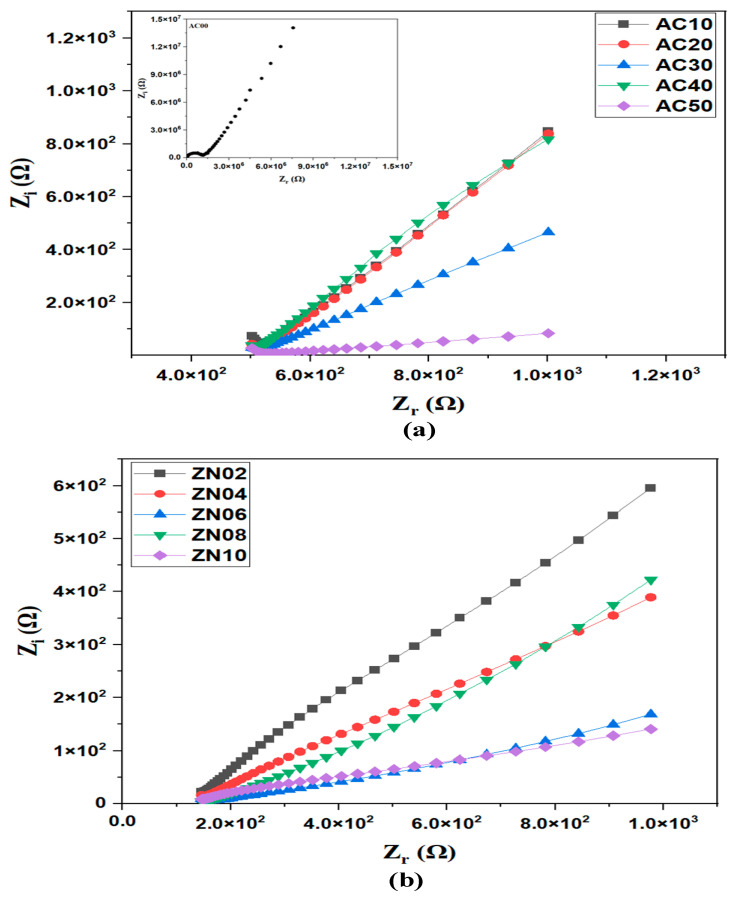
Room temperature Nyquist plots for (**a**) MC/PC/NH_4_Cl SPEs (**b**) MC/PC/NH_4_Cl/NCSPEs. Inset shows the Nyquist plot of MC/PC film.

A Nyquist plot (Z_i_ against Z_r_) was plotted for SPEs and NCSPEs, and the results are shown in Figure 6. In the Nyquist plot, Z_r_ values indicate the area of resistance and Z_i_ values describe the region of capacitance [35]. The conductivity of the synthesized electrolytes is determined by the following equation:

(1) $$\sigma =\frac{d}{R_{b}A}$$

where *d* represents the electrolyte thickness, *R* represents the bulk resistance, and *A* represents the electrode–electrolyte contact area. For measuring the thickness of all the SPEs prepared, a digital micrometer screw gauge was used. From our previous work [37], the ionic conductivity of pure MC/PC film was found to be 2.89 × 10^−9^ Scm^−1^. In this study, we realised a tremendous increase (order of 10^−4^) in conductivity of MC/PC films upon doping with various amount of NH_4_Cl. As can be seen from Table 1a, a room temperature ionic conductivity of 1.37 × 10^−5^ Scm^−1^ was observed when 10 wt.% NH_4_Cl was added to the host polymer. This was associated with the generation of charge-transfer complexes and a decreased crystallinity of the blend, as justified by Equation (2) [46,47];

(2) $$\sigma =F\sum n_{i}q_{i}\mu_{i}$$

In Equation (2), F represents the Faraday’s constant, whereas $n_{i}$, $q_{i}$, and $\mu_{i}$ indicate the number of available charge carriers, charge on an ion, and ion mobility, respectively. The equation shows that ionic conductivity is proportional to charge carrier concentration and ion mobility of the SPE. The ionic conductivity of the SPEs increased progressively with the addition of NH_4_Cl content, due to increase in carrier concentration, until an optimum value (6.43 × 10^−5^ Scm^−1^) was attained for the 50 wt.% doped sample (AC50).

Table 1b shows the variation of room temperature conductivity of AC50 with various amounts of ZnO nanofillers. As seen, the conductivity of the NCSPEs increased as the concentration of ZnO increased until an optimum conductivity of 3.13 × 10^−4^ Scm^−1^ was attained for ZN08. The increase in conductivity of the synthesized NCSPE sample might be attributable to the inclusion of nanosized ZnO nanofillers at a concentration up to 8 wt.%, where ZnO nanofiller acted as both a solid plasticizer and an electrolyte dissociation facilitator. Thus, the improved conductivity of the NCSPE might be related to the increase in amorphous nature brought about by the incorporation of ZnO nanofiller. In other words, improved conductivity resulting from the presence of ZnO nanofiller could be explained in terms of Lewis acid-base interactions. The interaction between Lewis’s acid sites of the nanofiller and Lewis base sites of both the polymer segments and NH_3_Cl^-^ anions increased the number of structural modifications of the polymer host. It also promoted proton-conducting pathways at the surface of the filler and lowered the ionic coupling (i.e., dissociation of ion pairs) between the H^+^ and NH_3_Cl^-^, thereby promoting salt dissociation and enhancing conductivity. In other words, the inclusion of additional ZnO nanofiller aided in lowering the bulk resistance of the NCPE. This was due to the fact that ZnO NP might prevent the recrystallization of excess NH_4_Cl salt and enlarge the amorphous phase domain, hence generating greater free volume.

Evidently, the conductivity of the optimised SPE decreased to 1.91 × 10^−4^ Scm^−1^, when 10 wt.% ZnO nanofillers were added. The reduction in conductivity might be due to the blocking impact of ion transport in the conduction routes, which might be induced by the generation of agglomerated ZnO nanocrystalline phase, which reduced the mobility of charge carriers for ionic conduction in the NCSPEs [48].

### 3.5. Dielectric Studies

#### 3.5.1. Dielectric Properties of MC/PC/NH_4_Cl SPEs

**Figure 7:**
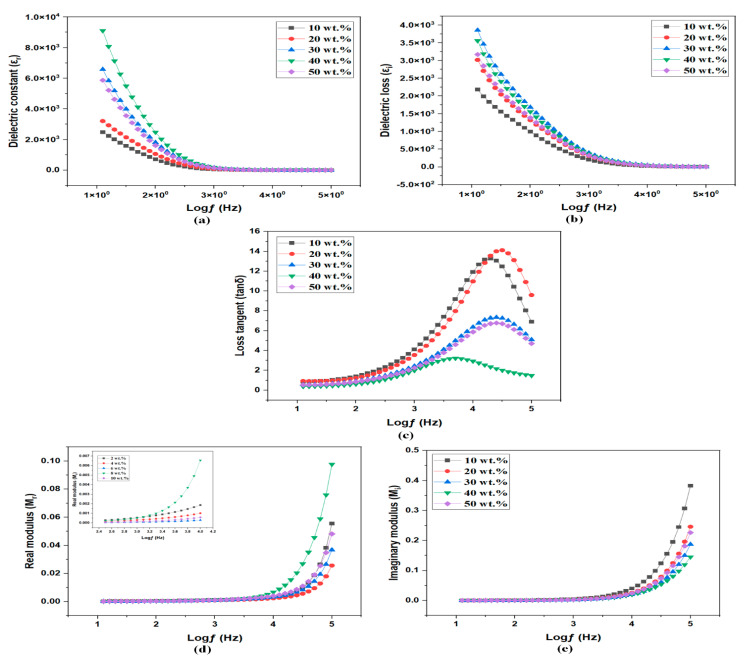
Dielectric spectra of MC/PC/NH_4_Cl films; (**a**) Real part of complex permittivity (**b**) Imaginary part of complex permittivity (**c**) Loss tangent (**d**) Real part of complex modulus (**e**) Imaginary part of complex modulus.

The ion conduction in the PE could be further examined using dielectric formalism, where the real part of the complex permittivity gives the measure of stored charges. Figure 7a shows the real part of complex permittivity, plotted as a function of frequency (10 Hz to 1 MHz). As seen, the plot is divided into the low-frequency part, where the dielectric constant is high and the high-frequency part where the dielectric constant is almost constant. Generally, there are two sources of dipoles; one is due to the dissociation of the salt and another is due to the dipoles in bulk [49]. When an electric field is applied, the dissociated ions accumulate at the electrode-electrolyte interface forming a double layer, thus leading to a high permittivity value at low frequencies [50]. At high frequencies, however, the ions fail to follow the field, and hence the dielectric constant attains constant values. The same is the case with the complex part of permittivity $\epsilon^{\prime \prime }$ (Figure 7b). The dielectric constant at low frequencies represents the carrier concentration (n) as given by Equation (3):

(3) $$n=n_{0}e^{-\frac{U}{k_{b}T\epsilon_{r}}}$$

where *ϵ* is the dielectric constant, and U is the salt dissociation energy.

As per EIS studies, the conductivity was linearly increasing with the salt concentration. However, the variation in the carrier concentration was not linearly varying with salt concentration (Figure 7a,b). This implied that carrier concentration and mobility greatly influence ionic conductivity as given by σ=neμ [51]. To further illustrate this, the real and imaginary part of complex permittivity at 10 Hz is given in Table 2. Furthermore, the plot of tangent loss as a function of frequency is given in Figure 7c. The tangent loss shows a characteristic maximum where the frequency of the rotating dipole matches that of the applied ac field. At this point, ωτ=1 from which relaxation time τ can be calculated. The relaxation time variation indicates a non-linear change in mobility. A similar observation was reported by Cyriac et al. [49]. It is worth noting that the highest conducting sample exhibited the highest relaxation time, implying lower mobility. However, the effect of lower mobility was balanced by the increased carrier concentration (as seen by the higher dielectric constant).

#### 3.5.2. Energy Modulus Spectra of MC/PC/NH_4_Cl SPEs

Figure 7d,e depict the variation of real $M^{\prime }$ and imaginary part $M^{\prime \prime }$ of complex modulus $M^{*}$ as a function of frequency. Modulus spectra formalism is advantageous over the dielectric formalism as it suppresses the electrode polarization at the low frequencies eliminating any false contribution to the dielectric constant [52]. Furthermore, both plots Figure 7d,e showed an increase in $M^{\prime }$ and $M^{\prime \prime }$ at the high-frequency side. The increase in modulus at high frequencies was attributed to the bulk effect of the resistance. However, well-defined dispersion peaks were not seen in the given frequency range. In contrast, long tails at the low-frequency side indicated the large capacitance due to ion accumulation leading to the electrode polarization, which confirmed non-Debye behaviour [53].

#### 3.5.3. Dielectric Properties of MC/PC/NH_4_Cl/ZnO NCSPEs

**Figure 8:**
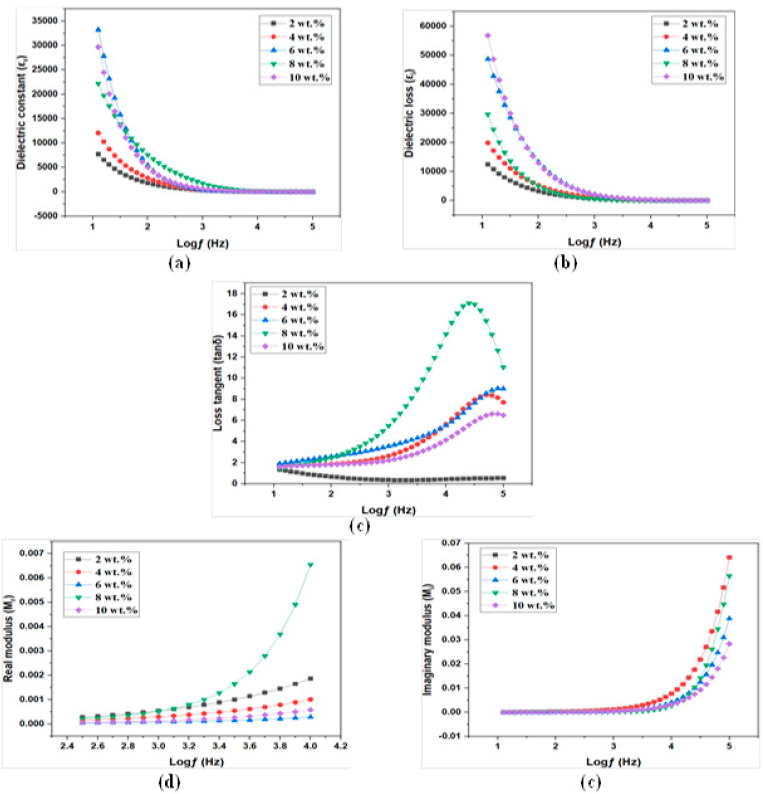
Dielectric spectra of MC/PC/NH_4_Cl/ZnO films; (**a**) Real part of complex permittivity (**b**) Imaginary part of complex permittivity (**c**) Loss tangent (**d**) Real part of complex modulus (**e**) Imaginary part of complex modulus.

The real and imaginary parts of complex permittivity were plotted for ZnO doped samples, and their variation with various concentrations of nanoparticles are shown in Figure 8a,b. As indicated earlier, the $\epsilon^{\prime }$ and $\epsilon^{\prime \prime }$ have high values at low frequencies and constant values at high frequencies and $\epsilon^{\prime }$ at low frequencies, representing carrier concentration. The values for the variation of real and imaginary parts of complex permittivity at 10 Hz with various amount of ZnO nanofiller concentration are given in Table 3.

When compared to ammonium salt doped samples, the dielectric constant of ZnO doped samples was very high. This enhancement in the dielectric constant with ZnO doping could be related to the increase in the amorphous nature of the polymer matrix. This observation supported the DSC analysis explained earlier, where the sample containing ZnO nanofiller exhibited the lowest *T_g_*. Furthermore, the enhancement in dielectric constant could also be interpreted in terms of the Lewis acid-base interaction between Lewis’s acid sites of ZnO and the Lewis base sites at polymer and anion of the salt. These interactions result in the structural modification in the polymer host and reduce the interaction between cation and anion of salt, leading to better salt dissociation [48]. However, at higher ZnO concentration (10 wt.%), the nanoparticles aggregated, leading to reduced carrier concentration, possibly due to ZnO agglomeration. 

Although the ionic conductivity varied linearly with nanoparticle concentration, reaching a maximum for ZN08, the carrier concentration was not highest for ZN08. Thus, we could assume that conductivity was strongly influenced not only by carrier concentration, but also by ion mobility. For better understanding, quantitative analysis of the variation in the parameters, such as carrier concentration (n) and mobility (μ), were attempted for ZnO doped samples and the results are discussed later in Section 3.5. The tangent loss spectra for various NCSPEs were plotted in Figure 8c. The relaxation time for the samples containing various amounts of ZnO nanofiller were calculated and the results are presented in Table 3. The lowest relaxation time was seen for 8 wt.% samples, indicating the highest ion mobility. A non-linear variation of the relaxation time was observed, which supported the fact that mobility varied in a non-linear fashion.

#### 3.5.4. Energy Modulus of MC/PC/NH_4_Cl/ZnO NCSPEs

Figure 8d,e show the variation of the real part $M^{\prime }$ and imaginary part $M^{\prime \prime }$ of complex modulus $M^{*}$ as a function of frequency for PE samples doped with various concentrations of ZnO nanoparticles. As indicated earlier, longer tails were observed at low frequencies, implying electrode polarization, and $M^{\prime }$ and $M^{\prime \prime }$ had higher values at higher frequencies, implying the bulk effect of resistance. Since there was no relaxation peak at higher frequencies in the given frequency range, further analysis of the relaxation phenomenon was not performed.

#### 3.5.5. Transport Properties of ZnO Doped Polymer Electrolytes

In order to investigate the transport parameters. such as carrier concentration (n), mobility (μ) and diffusion coefficient (D), as a function of ZnO concentration, Nyquist plots were fitted with the following equations, as given by Arof et al. [54]. The Nyquist plot which consists of the tilted spike at the low-frequency region and semicircle at high frequency region was fitted using Equations (4) and (5);

(4) $$Z_{r}=\frac{R+R^{2}k_{1}^{-1}\omega^{p_{1}}\cos\left(\frac{\pi p_{1}}{2}\right)}{1+2Rk_{1}^{-1}\omega^{p_{1}}\cos\left(\frac{\pi p_{1}}{2}\right)+R^{2}k_{1}^{-2}\omega^{2p_{1}}}+\frac{\cos\left(\frac{\pi p_{2}}{2}\right)}{k_{2}^{-1}\omega^{p_{2}}}$$

(5) $$\;Z_{i}=\frac{R^{2}k_{1}^{-1}\omega^{p_{1}}\sin\left(\frac{\pi p_{1}}{2}\right)}{1+2Rk_{1}^{-1}\omega^{p_{1}}\cos\left(\frac{\pi p_{1}}{2}\right)+R^{2}k_{1}^{-2}\omega^{2p_{1}}}+\frac{\sin\left(\frac{\pi p_{2}}{2}\right)}{k_{2}^{-1}\omega^{p_{2}}}$$

The parameters *R*, $k_{1}^{-1}$*,*$k_{2}^{-1}$, $p_{1}$, $p_{2}$ have the usual meaning as defined by Arof et al. [54]. The transport variables are calculated as follows.

(6) $$D=\frac{e{\left(k_{2}\varepsilon_{r}\varepsilon_{0}A\right)}^{2}}{\tau_{2}}$$

(7) $$\mu =\frac{eD}{k_{B}T}$$

(8) $$n=\frac{\sigma }{\mu e}$$

where $\varepsilon_{r}$, $k_{B}$ *T* and $\tau_{2}$ have the usual meaning. Table 4 listed the variation of $k_{2}^{-1}$, D, μ and n along with σ for prepared NCSPE samples.

Initially, the carrier concentration increased due to the incorporation of ZnO nanofiller. Due to limited space available for the carriers to move at higher carrier concentrations, the collision rate increased, thereby lowering the value of μ and D. At 8wt.% ZnO, the carrier concentration was reduced by two orders due to a lower dielectric constant. Hence, the collision rate reduced, leading to an increase in μ and D [55]. Thus, in the present system, the ionic conductivity of NCPE samples was not entirely dependent on carrier concentration, n. However, it was strongly associated with mobility, μ and the diffusion coefficient, D. These variations agreed with dielectric studies of NCPEs as justified by the dielectric spectra explained earlier. Similar results have been reported by other researchers as well [56,57].

### 3.6. LSV Analysis

**Figure 9:**
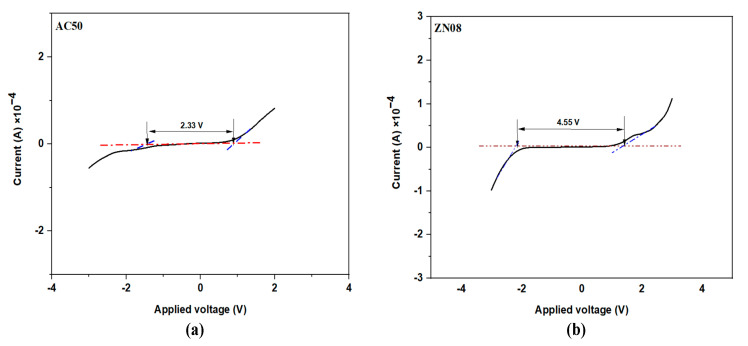
LSV plots of SPE and NCSPE (**a**) AC50 (**b**) ZN08.

For energy device engineering, it is necessary to evaluate the possible stability of PE systems. The LSV profile of the most conductive electrolyte, ZN08, at ambient temperature is presented in Figure 9. This will be used to evaluate the electrolyte’s breakdown voltage, a crucial characteristic that must be approximated for the operation of battery/EDLC technology [58]. For the AC50 sample (Figure 8a), the LSV curve reveals no apparent current density up to 2.33 V, indicating that the electrolyte’s electrochemical process is not occurring. However, when the potential exceeds 2.33 V, current density rises, which is progressively associated with electrolyte degradation [59]. The incorporation of ZnO nanofiller was observed (from Figure 8b) to improve the potential stability of the NCPE significantly. For the ZN08 sample, the breakdown voltage was obtained at around 1.75 V. However, the potential stability window of the ZN08 sample reached 4.55 V. The stability window is obtained by measuring the extent to which the SPE shows no sign of decomposition. At this point the applied current remains constant even as the voltage increases [9]. Interestingly, both AC50 and ZN08 electrolytes were ideal for use in applications because the threshold breakdown voltage demand for battery and EDLC systems was around 1.0 V [58]. Table 5 compares the potential stability values obtained in this study with some previous reports. As can be seen, this work presented more stable SPEs for potential application in solid state electrochemical devices.

## 4. Conclusions

Nanocomposite solid polymer electrolytes (NCSPEs) of MC/PC blend complexed with NH_4_Cl and ZnO nanofillers were synthesized via a solution cast approach. FTIR spectra were employed to verify the interactions and complexation of electrolyte constituents. The EIS results revealed that when the NH_4_Cl concentration increased from 10% to 50%, the bulk electrolyte’s resistance to charge transfer decreased, owing to an increase in charge carrier density. The electrolyte’s resistance further decreased with the addition of ZnO nanofiller from 2 wt.% to 8 wt.%, due to Lewis acid-base interactions between ZnO with MC/PC and NH_3_Cl^−^. The optimum sample exhibited ionic conductivity of 3.13 × 10^−4^ Scm^−1^, relaxation time of 6.03 × 10^−6^ and a stability window of 4.55 V. The distribution of relaxation time demonstrated that the electrolytes displayed non-Debye behaviour.

## Figures and Tables

**Table 1:** Upper half: Ionic conductivity of MC/PC/NH_4_Cl SPEs. Bottom half: Ionic conductivity of MC/PC/NH_4_Cl/ZnO NCSPEs.

**(a)**		
**Sample**	**Bulk Resistance × 10^2^ (Ohm)**	**Ionic Conductivity × 10^−5^ (Scm^−1^)**
AC10	5.31	1.37
AC20	4.34	2.13
AC30	2.65	3.68
AC40	2.41	4.53
AC50	1.84	6.43
**(b)**		
**Sample**	**Bulk Resistance × 10^2^ (Ohm)**	**Ionic Conductivity × 10^−4^ (Scm^−1^)**
ZN02	1.57	0.81
ZN04	1.31	1.02
ZN06	0.98	1.43
ZN08	0.46	3.13
ZN10	0.79	1.91

**Table 2:** Variation of real and imaginary parts of complex permittivity at 10 Hz and relaxation time as a function of salt concentration.

Sample	$\mathrm{Real}\;\mathrm{Part}\;\mathrm{of}\;\mathrm{Complex}\;\mathrm{Permittivity}\;(\epsilon^{\prime })$	$\mathrm{Imaginary}\;\mathrm{Part}\;\mathrm{of}\;\mathrm{Complex}\;\mathrm{Permittivity}\;(\epsilon^{\prime \prime })$	Relaxation Time τ (s)
AC10	2483.60	2181.01	7.97 × 10^−6^
AC20	3206.56	3017.85	5.03 × 10^−6^
AC30	6583.64	3857.87	6.34 × 10^−6^
AC40	9107.42	3557.13	3.18 × 10^−5^
AC50	5873.45	3173.41	6.34 × 10^−6^

**Table 3:** Variation of real and imaginary of complex permittivity at 10 Hz and relaxation time for polymer electrolyte sample as a function of ZnO concentration.

Sample	$\mathrm{Real}\;\mathrm{Part}\;\mathrm{of}\;\mathrm{Complex}\;\mathrm{Permittivity}\;(\epsilon^{\prime })$	$\mathrm{Imaginary}\;\mathrm{Part}\;\mathrm{of}\;\mathrm{Complex}\;\mathrm{Permittivity}\;(\epsilon^{\prime \prime })$	Relaxation Time τ (s)
ZN02	7696.06	12,379.27	-
ZN04	12,077.70	19,877.23	3.18 × 10^−6^
ZN06	33,229.64	48,698.14	2.01 × 10^−6^
ZN08	22,188.59	29,687.63	6.03 × 10^−6^
ZN10	29,687.63	56,848.16	2.01 × 10^−6^

**Table 4:** Summary of the parameters $k_{2}^{-1}$, *D*, *μ* and *n* for prepared samples.

Sample	$k_{2}^{-1}\;\left(F\right)$	σ (S/cm)	n (cm^−3^)	*μ* (cm^2^ V^−1^ s^−2^)	$D\;({\mathrm{cm}}^{2}\;s^{-1})$
ZN02	1.23 × 10^−4^	8.07 × 10^−5^	1.14 × 10^20^	5.47 × 10^−6^	1.42 × 10^−7^
ZN04	1.81 × 10^−4^	1.02 × 10^−4^	3.33 × 10^20^	2.22 × 10^−6^	5.76 × 10^−8^
ZN06	4.19 × 10^−4^	1.43 × 10^−4^	6.62 × 10^21^	1.32 × 10^−6^	3.43 × 10^−9^
ZN08	9.78 × 10^−5^	3.13 × 10^−4^	2.19 × 10^19^	1.05 × 10^−4^	2.72 × 10^−6^
ZN10	5.00 × 10^−4^	1.91 × 10^−4^	9.33 × 10^20^	1.03 × 10^−6^	2.66 × 10^−8^

**Table 5:** Comparison of selected SPEs based on electrochemical potential window.

Host Polymer	Salt	Potential Window (V)	Ref.
PEO	NH_4_I	1.09	[60]
CMC	NH_4_SCN	1.60	[61]
PVA/CS	NH_4_NO_3_	1.70	[62]
MC/Dex	NH_4_I	1.27	[36]
Dext/CS	NH_4_Br	1.54	[63]
MC/PC	NH_4_Cl	2.33	This work
PVA	NH_4_SCN/Glycerol	1.99	[64]
CS/MC	NH_4_NO_3_/Glycerol	1.87	[18]
PVA	NH_4_SCN/Proline	1.61	[65]
CS/MC	NH_4_I/Glycerol	2.20	[18]
PS/MC	NH_4_NO_3_/Glycerol	1.88	[66]
MC/PC	NH_4_Cl/ZnO NPs	4.55	This work

## Data Availability

The data presented in this study are available on request from the corresponding author. The data are not publicly available due to privacy.
